# Effect of Al Addition on Microstructure and Properties of CoCrNi Medium-Entropy Alloy Prepared by Powder Metallurgy

**DOI:** 10.3390/ma15249090

**Published:** 2022-12-19

**Authors:** Xuekun Ding, Jichang He, Jinde Zhong, Xiang Wang, Zhanjiang Li, Jun Tian, Pinqiang Dai

**Affiliations:** 1 College of Materials Science and Engineering, Fujian University of Technology, Fuzhou 350118, China; 2 Fujian Provincial Key Laboratory of Advanced Materials Processing and Application, Fuzhou 350118, China; 3 Fujian Metrology Institute, Fuzhou 350003, China; 4 College of Materials Science and Engineering, Fuzhou University, Fuzhou 350108, China

**Keywords:** medium-entropy alloys, microstructure, high-temperature resistance, mechanical properties

## Abstract

Powder metallurgy possesses the advantages of low energy consumption, less material consumption, uniform composition, and near-final forming. In order to improve the mechanical properties and high-temperature oxidation resistance of CoCrNi medium-entropy alloy (MEA), CoCrNiAl_X_ (X = 0, 0.1, 0.3, 0.5, 0.7) MEAs were prepared using mechanical alloying (MA) and spark-plasma sintering (SPS). The effect of aluminum content on the microstructure and properties of the MEAs was investigated. The results show that the CoCrNi MEA is composed of face center cubic (fcc) phase and some carbides (Cr_23_C_6_). With the increase in Al content, there exists Al_2_O_3_ precipitation. When the Al content is increased to Al_0.5_ and Al_0.7_, the body center cubic (bcc) phase begins to precipitate. The addition of aluminum significantly enhances the properties of the alloys, especially those containing fcc+bcc dual-phase solid solutions. The yield strength, compressive strength, and hardness of CoCrNiAl_0.7_ alloy are as high as 2083 MPa, 2498 MPa, and 646 HV, respectively. The high-temperature resistance also reaches the oxidation resistance level. Different oxides include Cr_2_O_3_, Al_2_O_3_, and (Co, Ni) Cr_2_O_4_ and NiCrO_3_ spinel oxides formed on the surface of alloys. The formation of an Al_2_O_3_ oxidation film prevents the further erosion of the matrix by oxygen elements.

## 1. Introduction

In 2004, Yeh et al. [1] proposed the concept of high-entropy alloys, which are different from traditional alloy design concepts. An alloy with a number of main elements greater than five and an atomic ratio of each main element between 5% and 35% is called a multi-component high-entropy alloy, for short, a high-entropy alloy. The configuration entropy of high-entropy alloys is greater than 1.5 R. According to this definition, a medium-entropy alloy is defined as an alloy whose configuration entropy is between 1.2 R and 1.5 R and consists of an equiatomic ratio of two to four principal elements [2,3]. Due to the mechanical properties, such as high strength, high plasticity, good low-temperature fracture toughness, and excellent corrosion resistance, of medium- and high-entropy alloys, they have received extensive attention in recent years [4,5,6,7,8,9,10,11,12]. Compared with other medium-entropy alloys and high-entropy alloys, CoCrNi medium-entropy alloys have excellent comprehensive properties, such as higher ductility, strength, and fracture toughness. For example, Gludovatz et al. [13] demonstrated that the tensile strength of CoCrNi medium-entropy alloy at room temperature is close to 1 GPa, and the fracture strain is as high as 70%. Compared with CrMnFeCoNi high-entropy alloy, it has higher ductility and strength [14].

At present, most researchers use the melting route to fabricate high/medium-entropy alloys. However, the melting route usually results in a coarse grain and composition segregation, which greatly limits its application in engineering [15]. In contrast, powder metallurgy is an important processing method of metal materials, and possesses the advantages of low energy consumption, less material consumption, uniform composition, fine gain size, and near-final forming. Many high- and medium-entropy alloys have been prepared using powder metallurgy. Moravcik et al. [16] fabricated isoatomic CoCrNi bulk MEAs using powder metallurgy. The alloy exhibited a high ultimate tensile strength of 1024 MPa and an elongation to fracture of 26%. The elastic modulus of the alloy reached 222 GPa and the thermal expansion coefficient (CTE) was measured as 17.4 × 10^−6^ K^−1^. Moazzen et al. [17] fabricated Fe_X_CoCrNi high-entropy alloys using mechanical alloying and spark-plasma sintering. It was found that the tensile strength increased from 480 MPa to 560 MPa, and the hardness increased from 320 HV to 400 HV. Moreover, the friction coefficient and weight loss decreased with the increase in Fe content.

Oxidation resistance is one of the most important properties of high- and medium-entropy alloys and has been studied by many groups. Generally, the addition of Al, Si, and Cr elements in the alloy will significantly enhance the oxidation resistance of the alloy. For example, Sudeep et al. [18] and Chen et al. [19] proposed that the addition of Al and Cr elements helps AlCoCrFeNi_2_ and Al_0.6_CrFeCoNiSi_X_ (X = 0, 0.3) high-entropy alloys to form a continuous oxide layer, which inhibits further oxidation. The addition of Si helps to promote the formation of a continuous oxide layer, which has a positive effect on the antioxidant behavior. The addition of V, Mo, and Ti elements will reduce the oxidation resistance of the substrate, which is not conducive to the improvement of the oxidation resistance of high-entropy alloys. For example, Cao et al. [20] realized that Al addition resulted in better oxidation resistance by forming a protective oxide scale, but Mo addition weakened its protective effect. Lu et al. [21] studied the effect of the addition of V and Mo elements on refractory high-entropy alloys and found that the addition of V would lead to catastrophic internal oxidation, and the addition of Ti would reduce the mass gain of the full coverage of the passive oxide scale, prolonging the passivation duration of the alloy.

As mentioned above, the CoCrNi MEA has excellent comprehensive properties compared with other alloys. The addition of Al is conducive to the formation of a continuous oxide layer of the alloy and enhances the oxidation resistance of the matrix. At present, there are few studies on the Al-containing CoCrNi MEA prepared using powder metallurgy. In this study, Al-added CoCrNi MEAs were prepared using powder metallurgy mechanical alloying, and the effects of different Al contents on the mechanical properties and oxidation resistance of CoCrNi MEAs were investigated. The results show that the mechanical properties and oxidation resistance of CoCrNi MEAs were improved greatly and the mechanisms for these improvements are discussed. This study paves the way for future applications in high-temperature fields.

## 2. Experimental Processures

Co, Cr, Ni, and Al metal powders with a purity of 99.9% were used as starting materials, and the average particle size was about 45 μm. The MEA powders CoCrNiAl_X_ (X represents atomic proportions, that is, X = 0, 0.1, 0.3, 0.5, and 0.7, which are denoted as alloys Al_0.1_, Al_0.3_, Al_0.5_, and Al_0.7_, respectively), were synthesized using mechanical alloying in a planetary ball mill (QM–QX4L, Changsha Mickey Instruments Co., Ltd., Changsha, China). The mechanical alloying process was carried out in a stainless steel bottle filled with high-purity argon gas. The ball-milling medium was cemented carbide balls with a ball-to-material ratio of 15:1, and the ball milling speed was 150 (±5) rpm/min. To avoid excessive cold welding, a process control agent (combined addition of ethanol and n-heptane) was added to the ball mill bottle. After ball milling for 30 h, the alloy powders were consolidated in a spark-plasma rapid-sintering furnace (SPS-5T-5-III, Shanghai Chenhua Technology Co., Ltd., Shanghai, China). The sintering parameters were sintering temperature of 1075 (±5) °C, holding time of 10 min, and pressure of 45 MPa. Subsequently, the alloy samples were cooled to room temperature in the furnace. The alloy samples were cut into 5 mm × 8 mm × 4 mm blocks, and then polished step by step with 600# and 3000# sandpaper, cleaned with acetone, dried, and ready for use.

The room-temperature compressive properties were measured on an Instron universal mechanical testing machine (LEGEND 2382) with a strain rate of 1 × 10^−3^ s^−1^. The sample was machined into a cylindrical shape with a diameter of 3.5 mm and a height of 5 mm. At least three compression tests were performed on each sample to obtain a reliable value. The hardness measurement of the bulk specimens was conducted on a Vickers hardness tester (HXD-1000T) with a load of 3000 gf (29.4 N) for 10 s. The reported hardness value was an average of at least 10 measurements. All experiments had good repeatability, and the error range is mentioned in Section 3.2. The high-temperature oxidation experiments were carried out using the thermostatic weight gain method with an air atmosphere at 1100 °C for 100 h. After taking out the samples every 20 h, the samples were weighed with a precision electronic balance (0.1 mg). The microstructure and composition of oxidation products and the cross-sectional morphology of the oxide film were analyzed using a Bruker D8 X-ray diffractometer (XRD, Karlsruhe, Germany) and a field emission scanning electron microscope (SEM, FEI-Nova Nano 450, Hillsborough, OR, USA) equipped with an energy dispersive spectrometer (EDS, Model Link-ISIS, Oxford, UK). The microstructure of the alloy was analyzed using an SEM and transmission electron microscope (TEM JEM-2100, 200 KV, Manufacturer, Tokyo, Japan).

## 3. Results and Discussion

### 3.1. Microstructure

**Figure 1:**
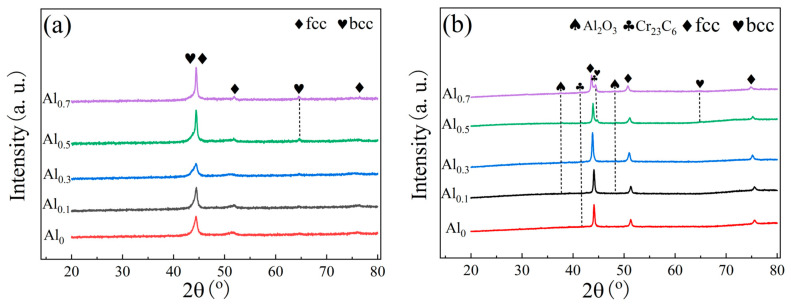
XRD patterns of powder and sintered alloy bulk: (**a**) powders after ball milling for 30 h; (**b**) sintered bulk alloys.

Figure 1a shows the XRD patterns of the alloy powders obtained by ball milling for 30 h. The elemental composition represented by each color in the figure has been marked on the left, and the dotted line represents the position of each phase in the XRD pattern. When the Al molar ratio is less than 0.5, the powder presents fcc single phase. When the Al molar ratio is greater than or equal to 0.5, the powder presents fcc+bcc dual phase. The grain refinement and high distortion energy of the powder after ball milling for 30 h led to an overlap in the broadening of the main peaks of fcc and bcc [22]. The XRD patterns of the SPS-sintered bulk alloy are shown in Figure 1b. When the Al molar ratio is less than 0.5 (i.e., alloys Al_0_, Al_0.1_, and Al_0.3_), the alloy exhibits a single-phase fcc phase structure. When the molar ratio of Al is greater than or equal to 0.5 (i.e., alloy Al_0.5_ and Al_0.7_), the structure of the alloy phase is dominated by the fcc phase, as well as a small amount of the bcc phase. This is consistent with the phase structure of the powder. From Figure 1b, with the Al content increases, the alloy gradually precipitates the bcc phase, and the diffraction peak intensity of bcc phase gradually increases, indicating that the increase in the Al content further leads to the increase in the bcc phase. This is consistent with the previously reported literature [23,24]. Al has a large atomic radius, and its addition to the alloy can cause severe lattice distortion, which increases the free energy of the system. The valence electron concentration (VEC) determines the lattice type. Since the VEC of bcc (≤6.87) is lower than that of fcc (≥8), the addition of Al promotes the transformation of fcc to bcc structure to reduce the lattice distortion energy [23,25]. Nevertheless, the main phase of the alloy is still the fcc phase. A carbide phase (Cr_23_C_6_) was also found in the XRD pattern of the Al_0.5_ alloy, which may be due to contamination with C present during sintering. Moreover, the formation of carbide is due to the strong affinity of Cr and C, which is consistent with the phenomenon seen in previous studies [26,27]. It is noteworthy that the alloy powder obtained by ball milling tends to adsorb oxygen. Therefore, the Al_2_O_3_ phase is easily generated during the sintering process [28], which makes the weak Al_2_O_3_ diffraction peaks appear in the XRD patterns.

**Figure 2:**
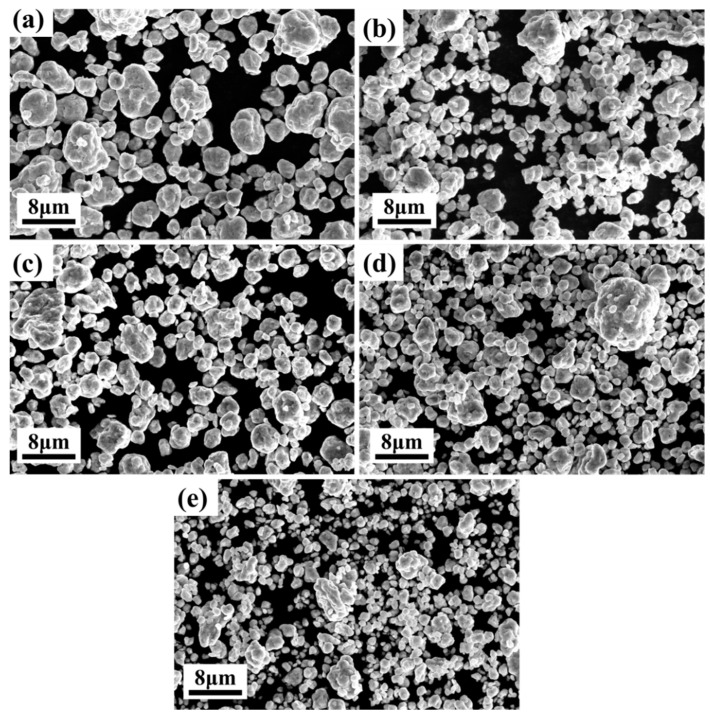
SEM images of different alloy powders after ball milling for 30 h: (**a**) Al_0_ alloy; (**b**) Al_0.1_ alloy; (**c**) Al_0.3_ alloy; (**d**) Al_0.5_ alloy; (**e**) Al_0.7_ alloy.

Figure 2 shows the SEM images of different alloy powders after ball milling for 30 h. During the mechanical alloying process, the powder undergoes repeated collision, deformation, cold welding, and pulverization, and finally reaches the equilibrium point between cold welding and pulverization, and the powder finally is a nearly spherical shape. Arbitrary powder particles were selected for point energy spectrum analysis, and the results are shown in Table 1. For the convenience of comparison, the nominal compositions of the various alloys are also listed in Table 1. It is found that the alloy composition is basically consistent with the design composition.

**Figure 3:**
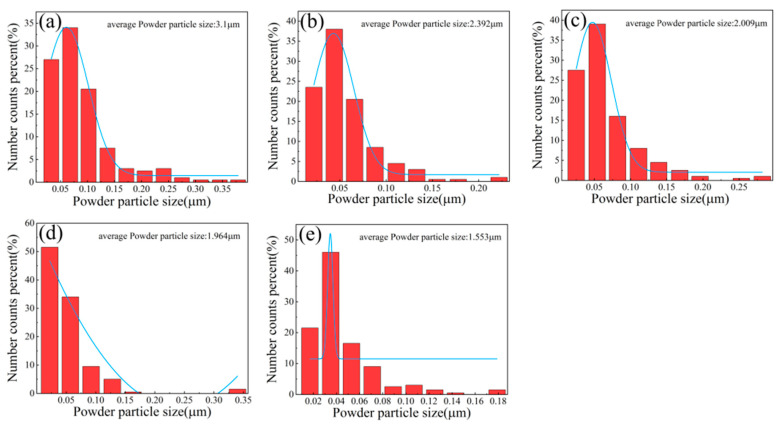
Particle size distribution of CoCrNiAl_X_ alloy powders milled for 30 h: (**a**) Al_0_ alloy powder, (**b**) Al_0.1_ alloy powder, (**c**) Al_0.3_ alloy powder, (**d**) Al_0.5_ alloy powder, (**e**) Al_0.7_ alloy powder.

Figure 3 shows the particle size distribution of CoCrNiAl_X_ alloy powders milled for 30 h. It can be seen that as the Al content increased from 0 to 0.7, the average powder particle size decreased from 3.1 to 1.553 um. With the increase in Al content, the plasticity of the alloy gradually decreases and the brittleness increases. Therefore, during the high-energy ball milling process, the alloy powder particles with higher Al content are easily broken into finer particles, which results in a gradual decrease in powder particle size.

**Figure 4:**
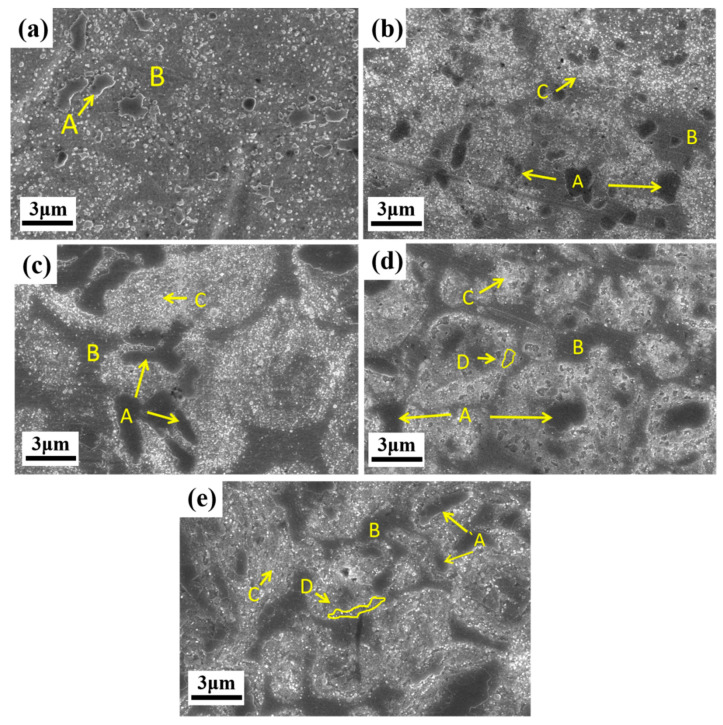
SEM morphology of sintered CoCrNiAl_X_ MEA: (**a**) Al_0_ alloy, (**b**) Al_0.1_ alloy, (**c**) Al_0.3_ alloy, (**d**) Al_0.5_ alloy, (**e**) Al_0.7_ alloy.

Figure 4 shows an SEM micrograph of the sintered alloys. The microstructure of the alloys exhibits bright contrast and dark contrast. Some dark regions appear in the Al_0_ alloy, and the dark regions gradually increase with the increase in Al content. The chemical composition of different regions in the alloy was analyzed by EDS and the results are listed in Table 2. The A area with obvious metallographic corrosion traces in Figure 4a contains more Cr and C elements. The composition distribution of area B is basically consistent with the nominal composition. Small and bright particles are evident in the C region, which has a higher Al content compared to the composition of the B region. The Al-Ni element segregation is observed in the D region of Figure 4d,e. Combined with the XRD results in Figure 1b, the A region is the carbide region, the B region is the matrix fcc, and the C and D regions are the Al_2_O_3_ segregation region and the bcc phase formation region, respectively. The bcc phase in the Al_0.5_ alloy is composed of dispersed particles. With the increase in Al concentration, the precipitated bcc phase particles begin to increase. Subsequently, the dispersed particles are connected into larger groups, and the precipitation positions are mostly distributed in the region of Al elemental segregation, as shown in Figure 4e.

**Figure 5:**
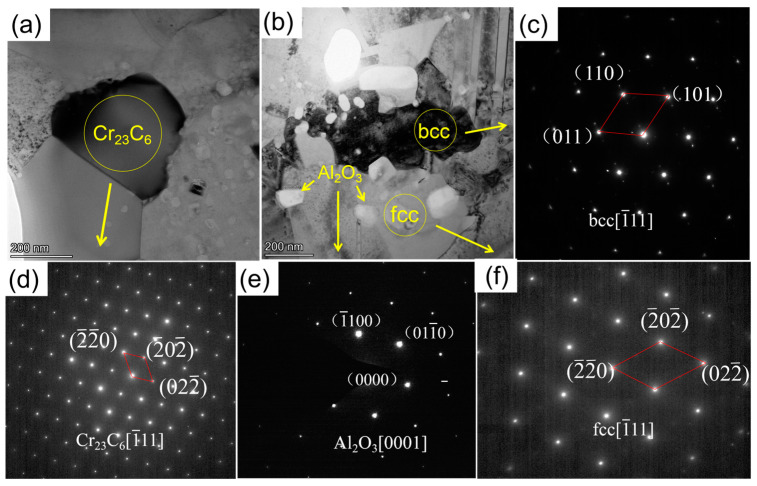
TEM images of Al_0.5_ bulk alloy: (**a**,**b**) high-magnification bright-field images, (**c**–**f**) the SAED patterns of the grains.

The high-magnification microstructure of the bulk alloy was revealed by TEM/EDS. The TEM bright-field image and Selected Area Electron Diffraction (SAED) patterns of the Al_0.5_ alloy are shown in Figure 5. Figure 5a,b are high-magnification bright-field images of the Al_0.5_ alloy. Figure 5b,c show SAED patterns of different grains. In addition to the fcc phase and bcc phase, there are carbides in the Al_0.5_ alloy. This is confirmed by the SAED patterns along the [$\bar{1}11$] zone axis in Figure 5c,d,f corresponding to areas A, B, and D, respectively, in Figure 4. Figure 5e shows the SAED pattern of Al_2_O_3_ grains along the [0001] zone axis. According to the chemical composition analysis in Table 3, the small white particles are Al_2_O_3_, which is also consistent with the above speculation.

### 3.2. Mechanical Properties

**Figure 6:**
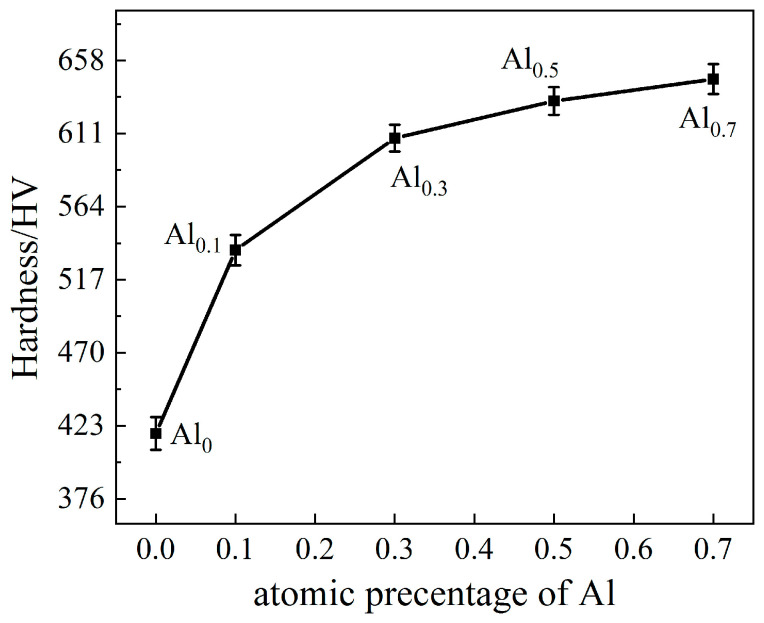
Variation of Vickers hardness with Al content in the alloys.

**Figure 7:**
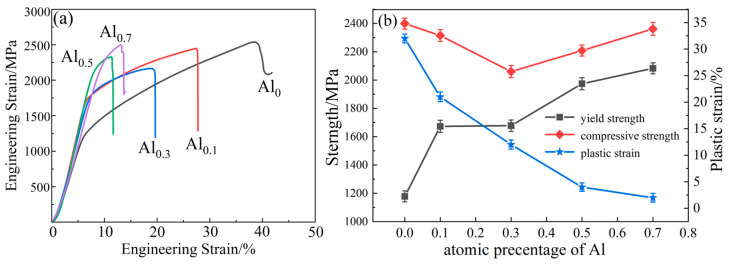
Compressive mechanical properties of the alloys as a function of Al content: (**a**)Compressive stress–strain curve; (**b**) yield strength, compressive strength, and plastic strain.

The relationship between hardness and Al content is shown in Figure 6. The hardness of the alloy increases with the increase in Al content. The hardness of Al_0.7_ alloy (646 ± 9.86 HV) is 51% higher than that of the Al_0_ alloy (429 ± 6.27 HV). The compressive engineering stress–strain curve of the alloy is shown in Figure 7a. The compressive strength, yield strength, and plastic strain as a function of Al content are shown in Figure 7b. The Al_0_ alloy has the lowest yield strength but better plasticity with a plastic strain of 32 ± 4%. Surprisingly, the addition of Al increases the yield strength from 1178 ± 12.54 MPa (Al_0_ alloy) to 2083 ± 18.11 MPa (Al_0.7_ alloy), an increase of 77%. However, the plastic strain decreased from 32 ± 4% to 2 ± 0.3%. The compressive strength first decreases and then increases with the increase in Al content, and the compressive strength of Al_0.7_ is the highest (2498 ± 25.64 MPa).

From Figure 7, it can be seen that the CoCrNi MEA is significantly strengthened by the addition of Al elements. In particular, the yield strength almost doubled. For Al_0_, Al_0.1_, and Al_0.3_ alloys, in addition to fcc solid solution phase and carbide phase, there is also an Al_2_O_3_ phase. Hard particles are distributed in the alloy and can hinder the movement of dislocations during plastic deformation [29]. Therefore, a certain precipitation-strengthening effect can be produced. As shown in the TEM images in Figure 5a,b, carbide particles are precipitated on the grain boundaries, and Al_2_O_3_ particles are distributed in the interior grain and grain boundaries. In addition, through EDS analysis, it can be found that there are Al elements present in the fcc phases (Figure 4, Table 2). Since Al has a large atomic radius, it is solid-dissolved in the fcc phase, which can produce a solid-solution strengthening effect [23]. Therefore, compared with the Al_0_ alloy, the main strengthening mechanisms of Al_0.1_ and Al_0.3_ alloys should be solid-solution strengthening caused by Al solute atoms and precipitation strengthening caused by the hard-particle phase. For Al_0.5_ and Al_0.7_ alloys, the bcc solid solution phase is formed in the alloy, while the main phase remains the fcc phase. The close-packed plane of the bcc structure is {110}, and the slip direction of each slip surface is less than that of the fcc structure, which makes it more difficult to slip. In addition, alloys with bcc structure have higher hardness but poor plasticity, while alloys with fcc structure have low strength but good plasticity [30,31]. Therefore, Al_0.5_ and Al_0.7_ alloys are mainly due to precipitation strengthening and solid-solution strengthening. The combination effect of solution strengthening and bcc phase leads to an increase in yield strength and hardness.

**Figure 8:**
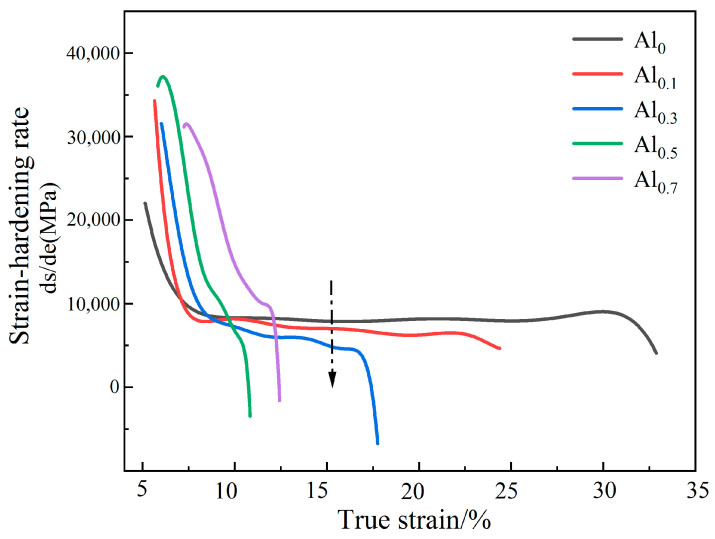
Strain hardening rate (dσ/dε) and true strain curves of alloys.

From the morphology in Figure 4 and the TEM image in Figure 5, it can be seen that a large number of Al_2_O_3_ particles and carbide particles are distributed in the grains and grain boundaries, which may hinder the movement of dislocations. The larger the fcc lattice distortion caused by the dissolution of the macroatom Al element, the greater the resistance to dislocation motion [25,32]. It may lead to dislocation accumulation, stress concentration, crack initiation, plastic instability, and ultimately material fracture. This can also be seen from the strain hardening rate (dσ/dε) versus true strain curve of the alloy in Figure 8. In addition, the strain-hardening exponents of the different alloys are shown in Table 4. It can be seen from the figure that under the same strain, with the increase in Al content, the strain hardening ability of the Al_0_, Al_0.1_, and Al_0.3_ alloys gradually decreases, and the required flow stress also decreases gradually, so the compressive strength and plasticity decrease continuously. Therefore, the compressive strength and plasticity of Al_0_, Al_0.1_, and Al_0.3_ alloys decrease simultaneously with the increase inof Al content. When the content of Al is increased to 0.5, it is worth noting that the compression curves of Al_0.5_ and Al_0.7_ alloys in Figure 7a exhibit obvious work-hardening characteristics, indicating the precipitation of bcc phase, resulting in an increase in strength and decrease in plasticity.

### 3.3. Oxidation Resistance

#### 3.3.1. The Oxidation Kinetics

**Figure 9:**
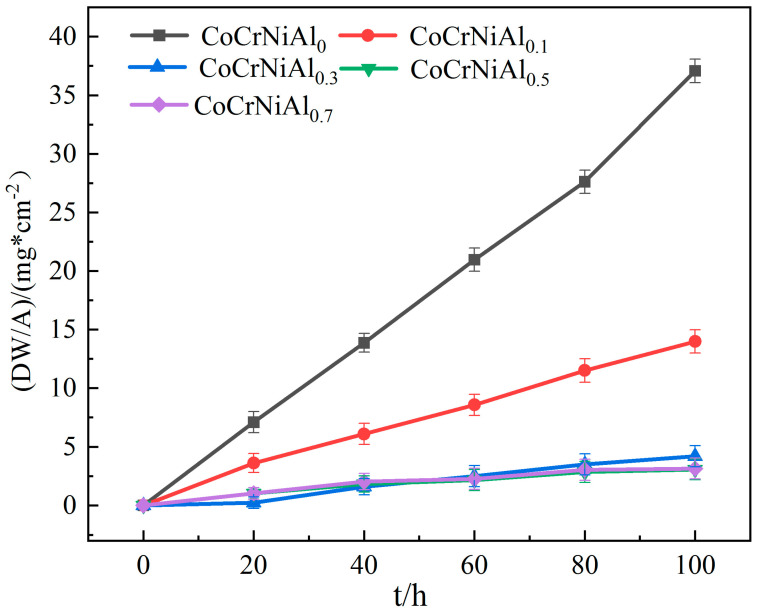
CrCoNiAlx Oxidation kinetics curves of medium-entropy alloys at 1100 °C.

The CoCrNi MEAs with Al addition are basically not oxidized below 1100 °C and their weight gain is too little to weigh. Therefore, the oxidation tests of the CoCrNiAl_X_ MEAs were only carried out at 1100 °C. Figure 9 shows the oxidation kinetics of the CoCrNiAl_X_ MEAs at 1100 °C. All the oxidation kinetics curves are nearly straight lines. With the prolongation of oxidation time, the oxidation weight gain of the five alloys increased gradually. With the gradual increase in Al content, the oxidation weight gain of the alloys decreases, indicating that the increase in Al content improves the oxidation resistance of the alloy at high temperatures. Combined with the observation of the oxidation process of the alloy, it is found that the oxide spallation phenomenon occurs in the Al_0_ and Al_0.1_ alloys after the first 10 h of high-temperature oxidation at 1100 °C. The oxide scar peels off to reveal a new metal surface, further deepening the oxidation. With the increase in the Al content, the exfoliation gradually decreases until it disappears.

The oxidation weight gain rates of the five MEAs in air at 1100 °C are Al_0_ > Al_0.1_ > Al_0.3_ > Al_0.5_ > Al_0.7_. After 100 h, the oxidation weight gain values of entropy alloys in Al_0_, Al_0.1_, Al_0.3_, Al_0.5_ and Al_0.7_ are shown in Table 5. During the oxidation process, the oxidation weight gain of the Al_0.5_ MEA is close to that of the Al_0.7_ MEA, and the oxidation performance is better, while the oxidation resistance of the Al_0_ alloy is relatively poor. All alloys exhibit an initial transient oxidation followed by varying degrees of linear oxidation growth. It can be observed that the oxidation rate constant drops sharply when the addition of Al starts (Table 5). It is further proved that the addition of Al delays the alloys’ oxidation at high temperatures.

#### 3.3.2. Microstructure of the Oxidated Layers

**Figure 10:**
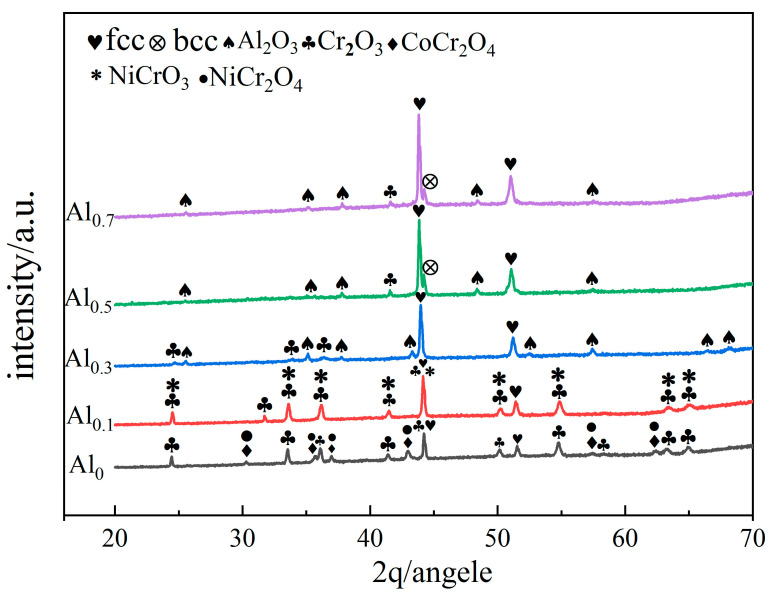
XRD patterns of surface oxidation products of CoCrNiAl_X_ MEAs at 1100 °C.

The XRD patterns of the surface oxidation products of CoCrNiAl_X_ alloys at 1100 °C are shown in Figure 10. The elemental composition represented by each color in the figure has been marked on the left. Oxidation products are formed on the surface of CoCrNiAl_X_ alloys after 100 h oxidation. The surfaces of Al_0_ and Al_0.1_ alloys contain Cr_2_O_3_ and spinel oxides NiCrO_3_, NiCr_2_O_4_, and CoCr_2_O_4_. The surfaces of Al_0.3_, Al_0.5_, and Al_0.7_ mainly contain two oxides, Al_2_O_3_ and Cr_2_O_3_. According to the XRD phase analysis, after high-temperature oxidation at 1100 °C for 100 h, the Al_0_ and Al_0.1_ alloys have fcc phase diffraction peaks, which is mainly due to poor adhesion and excessive peeling of the Cr_2_O_3_ oxide layer, resulting in the exposure of the fcc phase matrix. The surfaces of the Al_0.5_ and Al_0.7_ alloys have not only the characteristic peaks of the matrix fcc but also the diffraction peaks of the bcc. This phenomenon indicates that the thickness of the oxide layer formed by the three alloys at this oxidation temperature is relatively thin, resulting in the XRD rays penetrating the oxide layer to make the fcc phase detected. The appearance of the matrix fcc phase in the alloy and the increasing intensity of the diffraction peaks indicate that the addition of Al improves the oxidation resistance of the alloy greatly. Some researches have shown [33] have shown that Al can also react selectively with N at high temperature. However, this phenomenon was not found in this paper. This may be due to the different preparation methods which cause differences in microstructure.

**Figure 11:**
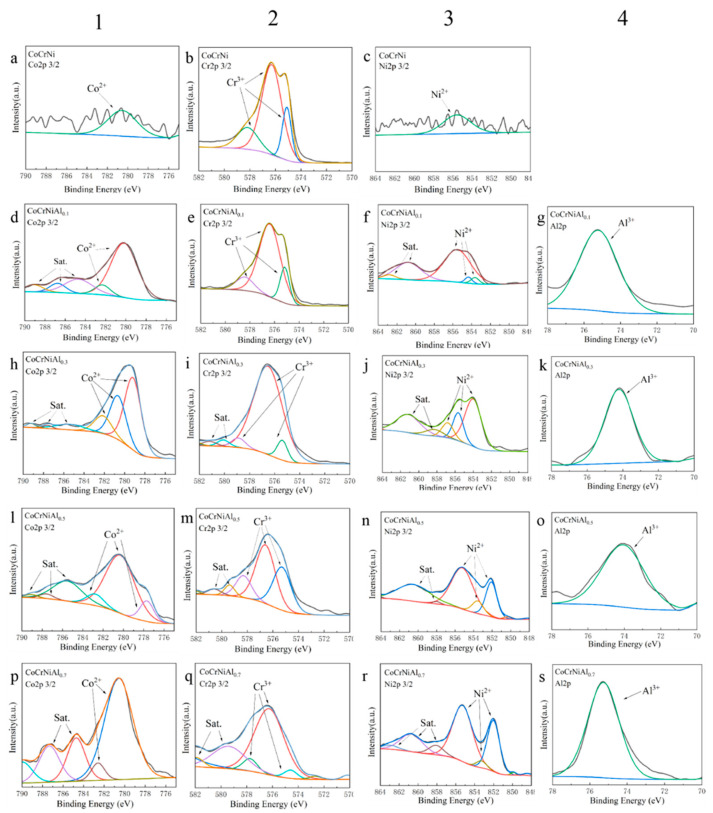
XPS Analysis of Oxidized Surface: (**a**–**c**) CoCrNiAl_0_;(**d**–**g**) CoCrNiAl_0.1_; (**h**–**k**) CoCrNiAl_0.3_; (**l**–**o**) CoCrNiAl_0.5_; (**p**–**s**) CoCrNiAl_0.7._

In order to confirm the formation mechanism of high-temperature oxides of CoCrNiAl_X_ alloys, the oxidized surface of the alloys after oxidation at 1100 °C for 100 h were analyzed by XPS and the results are shown in Figure 11. All four elements exist in relatively stable valence states (Co^2+^, Cr^3+^, Ni^2+^, Al^3+^) on the oxidized surface, and the XPS spectra of the core levels were fitted to the peaks using Thermo Advantage. Combining the XRD analysis results with XPS, it can be seen that Cr_2_O_3_ and NiCrO_3_ correspond to the Cr^3+^ peaks in the schematic diagram of the second column in Figure 11. CoCr_2_O_4_ and NiCr_2_O_4_ match the peaks of Co^2+^ and Ni^2+^ in the schematic diagrams in the first and third columns of Figure 11, respectively. Al_2_O_3_ corresponds to the single peak of Al^3+^ in the schematic diagram in the fourth column of Figure 11. The above results show that the analysis results of XPS are consistent with the results of XRD, that is, after oxidation at 1100 °C for 100 h, the surface oxides exist in the form of Cr_2_O_3_, Al_2_O_3_, and stable metal oxo-acid salts. The results of XPS further prove that when the Al content is low, the Cr element first undergoes selective oxidation, forming Cr-containing oxides attached to the surface of the alloy; while when the Al content gradually increases, Al firstly undergoes selective oxidation, mainly forming Al_2_O_3_, which covers the surface of the alloy to avoid further oxidation by oxygen elements [34].

**Figure 12:**
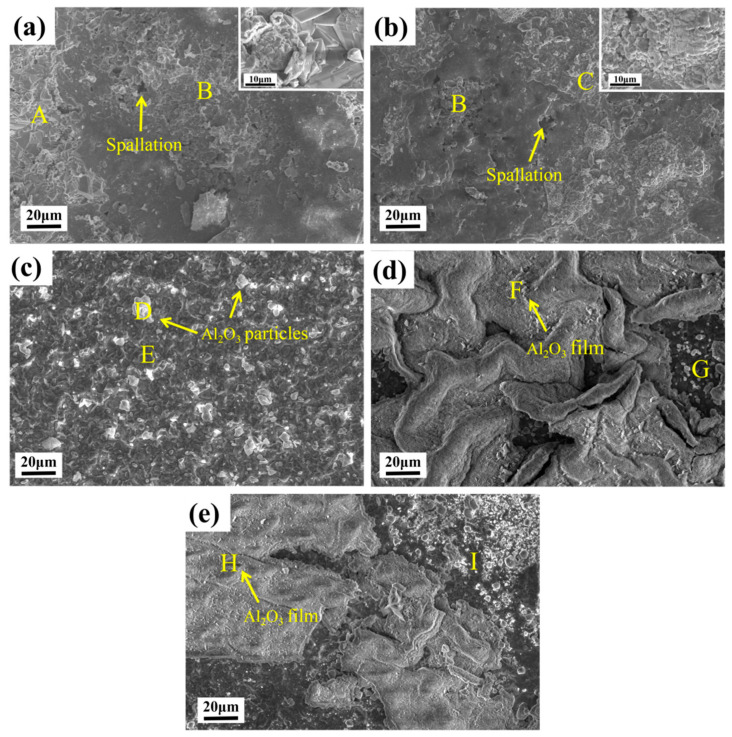
SEM images of oxidized surfaces of CoCrNiAl_X_ (X = 0, 0.1, 0.3, 0.5, 0.7) medium-entropy alloys at 1100 °C for 100 h: (**a**) CoCrNiAl_0_; (**b**) CoCrNiAl_0.1_; (**c**) CoCrNiAl_0.3_; (**d**) CoCrNiAl_0.5_; (**e**) CoCrNiAl_0.7._

The surface oxidation morphology of CoCrNiAl_X_ (X = 0, 0.1, 0.3, 0.5, 0.7) is shown in Figure 12. It can be seen that there is a clear difference among the alloys. As shown in Figure 12a,b, the oxide layer of the Al_0_ and Al_0.1_ alloys has a loose hollow structure, resulting in large-scale spalling on the oxidized surface, as shown by the yellow arrows in the picture. The insets in Figure 12a,b are the magnified morphology of regions A and C. Regions A and C are regions where spinel phases exist. Combining the chemical compositions of different regions of the oxidized surface in Table 6 and the XRD analysis of the surface oxidation products in Figure 10, spinel phases dominated by (Co, Ni) Cr_2_O_4_ and NiCrO_3_ were formed. The B area contains more Cr and O elements, mainly Cr_2_O_3_. As the Al content increases, Al_2_O_3_ particles begin to appear in the D area, and then a dense Al_2_O_3_ film begins to form, preventing further oxidation of the matrix by oxygen, and the spalling situation is alleviated. A large number of Al_2_O_3_ particles is formed on the surface of the Al_0.3_ alloy, as shown in Figure 12c, indicating that Al diffuses rapidly from the matrix to the surface of the oxide layer. The E, G, and I regions are the regions where Cr_2_O_3_ and the matrix phase exist according to Table 6. The F and H regions are film, which are Al_2_O_3_ phases in combination with the XRD analysis in Figure 10. As the Al content increases to Al_0.5_ and Al_0.7_, under the action of high temperature, a dense but discontinuous alumina film is formed, which effectively prevents further oxidation of the matrix.

**Figure 13:**
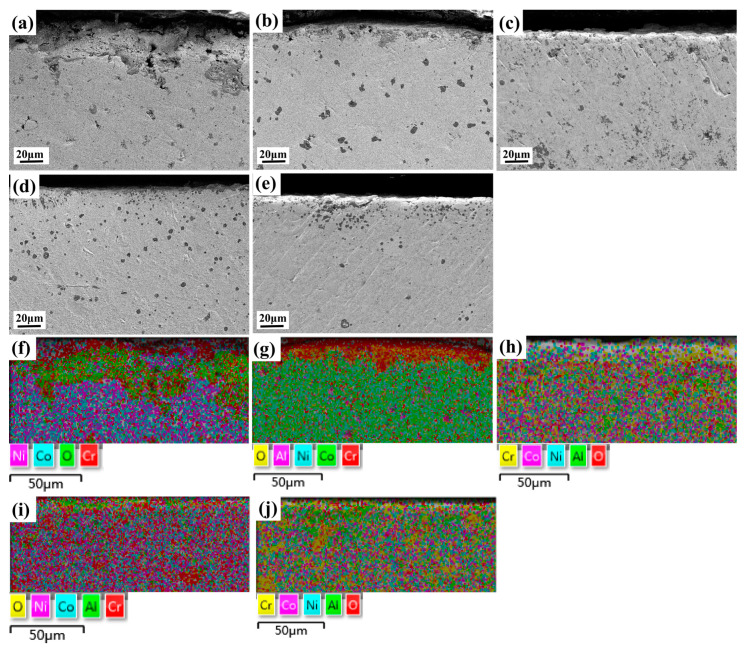
Cross-sectional SEM images of CoCrNiAl_X_ (X = 0, 0.1, 0.3, 0.5, 0.7) MEAs and cross-sectional EDS scans after oxidation at 1100 °C for 100 h: (**a**,**f**) CoCrNiAl_0_; (**b**,**g**) CoCrNiAl_0.1_; (**c**,**h**) CoCrNiAl_0.3_; (**d**,**i**) CoCrNiAl_0.5_; (**e**,**j**) CoCrNiAl_0.7._

The SEM section image of CoCrNiAl_X_ oxidized at 1100 °C for 100 h is shown in Figure 13. When the Al content is low, the oxidation depth is large and the structure is loose, resulting in a rougher cross-section of the oxidized substrate-oxide film. The selective oxidation of Al and Cr elements with oxygen occurs due to their high affinity for the element [35,36,37]. In order to further determine the sequences and products of oxidation, the Gibbs free energy of the products of several oxidation at 1100 °C was calculated using the formula shown in [38]. The formula is as follows:

(1) $$△\emptyset_{T}=\sum {\left(n_{i}\emptyset_{i},T\right)}_{product}-\sum {\left(n_{i}\emptyset_{i},T\right)}_{reac\;tan\;t}$$

(2) $$△G_{T}^{\theta }=△H_{T_{0}}^{\theta }-T△\emptyset_{T}\;\;$$

**Figure 14:**
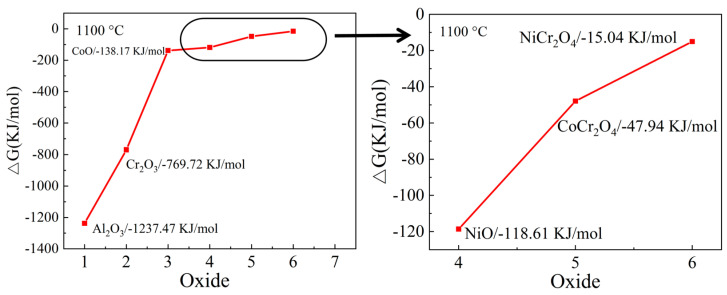
Gibbs free energy at 1100 °C of oxides that may be formed during oxidation.

*n_i_* is the amount of substance i in each substance participating in the reaction, ∅*_i_* is the Gibbs free energy of substance i in each substance participating in the reaction, and the values of each parameter can be obtained from [39]. *T* represents the reaction temperature. $△\emptyset_{T}\;$ is the reaction Gibbs free energy function, $△G_{T}^{\theta }$ is the Gibbs free energy, and $△H_{T_{0}}^{\theta }$ is the relative reaction enthalpy difference. The calculation results are shown in Figure 14. It can be seen that the Gibbs free energy values of the oxidation products Al_2_O_3_ and Cr_2_O_3_ at 1100 °C are −1237.47 KJ/mol and −769.72 KJ/mol, respectively, which are the lowest among all oxidation products. This result indicates that Al and Cr selective oxidation occurs first, and the reaction is also spontaneous.

In contrast, the Al_0_ alloy does not contain Al, but only Cr, which makes it difficult to form a dense oxide layer. According to the EDS mapping of the oxidation cross-section shown in Figure 13f, it can be seen that the oxide layer is mainly composed of loose Cr_2_O_3_, and the spinel phase of (Co, Ni) Cr_2_O_4_ is attached to its outer layer. The poor adhesion of Cr_2_O_3_ results in continuous peeling during the oxidation process [36,37]. A dense oxide film cannot be formed, so the oxidation process is mainly the oxidation of the rate-controlled gas–metal interface reaction [38]. Other elements in the matrix diffuse into the outer layer from the voids generated by shedding, thereby forming the spinel phase of (Co, Ni) Cr_2_O_4_ and NiCrO_3_. The Al_0.1_ alloy contains less Al, so the existence of Al_2_O_3_ cannot be detected in XRD. Combined with the EDS mapping of the oxidation cross-section shown in Figure 13g, its oxidation process mainly depends on the diffusion of Cr, and the oxidation mechanism is the same as that of the Al_0_ alloy. When the Al content increases to 0.3, no obvious segregation of elements such as oxygen is found in Figure 13h–j. As shown in Figure 12, Al_2_O_3_ particles begin to form on the surface of the Al_0.3_ alloy. Under the action of high temperature, a dense but discontinuous Al_2_O_3_ film is formed on the surface of the Al_0.5_ and Al_0.7_ alloys, which blocks the intrusion of oxygen elements and avoids further oxidation of the matrix.

During the oxidation process, the diffusion of Al and Cr plays a key role, and the slow diffusion may also be partly responsible for the apparent stability of HEA [40]. The high temperature causes a slow and steady diffusion of Al and Cr elements from the matrix to the surface of the oxide scale, and then reacts with oxygen to form oxides.

## 4. Conclusions

The CoCrNiAl_X_ MEAs were prepared by mechanical alloying and SPS sintering. The microstructure, mechanical properties, and high-temperature oxidation resistance of the alloys were evaluated. The main conclusions are as follows:

1. When the Al content is less than 0.3, the sintered CoCrNiAl_X_ MEA is mainly composed of fcc single-phase solid solution and Cr_23_C_6_ carbide; when the Al content is greater than or equal to 0.5, CoCrNiAl_X_ MEA is composed of fcc+bcc dual-phase solid solution phase, Cr_23_C_6_ carbide, and Al_2_O_3_ phase.
2. The addition of Al has a significant strengthening effect. As the molar ratio of Al increases from 0.1 to 0.7, the yield strength is increased from 1178 MPa to 2083 MPa, which is nearly doubled. The plastic strain is decreased from 32% to 2%. The hardness is increased from 429 HV to 646 HV.
3. The strengthening mechanisms of Al_0_, Al_0.1_, and Al_0.3_ alloys are mainly due to solid-solution strengthening and precipitation strengthening; Al_0.5_ and Al_0.7_ alloys are mainly a combination of solid-solution strengthening, precipitation strengthening, and bcc phase strengthening.
4. At 1100 °C, the oxidation resistance of the CoCrNiAl_X_ MEAs increased with the Al content. The presence of Cr_2_O_3_, Al_2_O_3_, and other spinel oxides was verified during oxidation. The element Al plays an important role in improving high-temperature oxidation resistance and the formation of an Al_2_O_3_ oxidation film prevents further erosion of the matrix by oxygen elements. Al_0_ and Al_0.1_ alloys have lower oxidation resistance, while Al_0.3_, Al_0.5_, and Al_0.7_ alloys have stronger oxidation resistance.

## Figures and Tables

**Table 1:** EDS analysis results of CoCrNiAl_X_ medium-entropy alloy powder after ball milling for 30 h.

Alloys	Compare	Co/%	Cr/%	Ni/%	Al/%
Al_0_	Nominal	33.3	33.3	33.3	0
	Actual	35.3	33.6	31.1	0
Al_0.1_	Nominal	32.3	32.3	32,3	3
	Actual	38.6	28.5	30.0	2.9
Al_0.3_	Nominal	30.3	30.3	30.3	9
	Actual	30.8	26.1	32.1	11.0
Al_0.5_	Nominal	28.6	28.6	28.6	14
	Actual	30.5	26.0	28.9	14.6
Al_0.7_	Nominal	27	27	27	19
	Actual	30.1	28.4	24.2	17.3

**Table 2:** EDS analysis results of the bulk alloy after SPS sintering (area shown in Figure 4).

Alloys	Regions	Co/%	Cr/%	Ni/%	Al/%	C/%
Al_0_	A	6.4	52.8	3.8	0	36.9
	B	31.1	36.3	32.6	0	0
Al_0.1_	A	3.9	54.2	2.3	0.7	38.8
	B	33.7	30.6	34.6	1.1	0
	C	33.1	29.4	32.3	5.2	0
Al_0.3_	A	9.3	56.0	8.6	2.1	24.0
	B	29.8	33.3	29.6	7.3	0
	C	31,6	26.8	30.3	11.4	0
Al_0.5_	A	3.9	54.9	2.2	0.1	38.9
	B	30.3	26.8	31.9	11.0	0
	C	27.8	26.8	25.9	19.4	0
	D	9.4	8.9	43.3	38.3	0
Al_0.7_	A	6.2	53.4	3.5	2.2	34.7
	B	35.7	27,7	27.2	9.3	0
	C	31.0	23.2	24.2	21.6	0
	D	10.9	6.9	44.3	37.9	0

**Table 3:** TEM/EDS analysis results of the bulk alloy after SPS sintering (region shown in Figure 5).

Element	Co/%	Cr/%	Ni/%	Al/%	C/%	O/%
fcc	31.3	29.5	33.9	5.3	0	0
bcc	8.52	5.68	41.3	44.5	0	0
Cr_23_C_6_	4.51	77	2.79	0	15.7	0
Al_2_O_3_	0.91	1.46	0.63	35.8	0	61.2

**Table 4:** Strain hardening exponents of different alloys.

	Al_0_	Al_0.1_	Al_0.3_	Al_0.5_	Al_0.7_
strain hardening exponents	0.622	0.406	0.339	0.441	0.555

**Table 5:** CoCrNiAlx MEAs Oxidation weight gain values and Oxidation rate constant.

	CoCrNiAl_0_	CoCrNiAl_0.1_	CoCrNiAl_0.3_	CoCrNiAl_0.5_	CoCrNiAl_0.7_
Oxidation rate constant (Kp/g^2^.cm^−4^.s^−1^) (1 × 10^−9^)	1.9 ± 0.3	2.76 ± 0.026	2.45 ± 0.0028	1.25 ± 0.0025	1.33 ± 0.0018
Oxidation weight gain (mg.cm^−2^)	37.079 ± 4.92	13.988 ± 2.83	4.186 ± 1.67	3.070 ± 0.79	3.147 ± 0.87

**Table 6:** EDS analysis results of the oxidized surface of the alloy (area shown in Figure 12).

Alloys	Regions	Co/%	Cr/%	Ni/%	Al/%	O/%
Al_0_	A	6.7	29.3	4.4	0	59.6
	B	3.0	32.7	4.9	0	59.4
Al_0.1_	B	0	52.3	0	0	47.7
	C	0.8	46.6	13.2	0	38.4
Al_0.3_	D	4.5	3.8	4.2	45.9	41.6
	E	6.6	58.0	5.2	4.3	25.9
Al_0.5_	F	0	1.2	0	45.7	53.2
	G	8.9	39.5	7.6	7.5	36.5
Al_0.7_	H	0	3.2	0	57.7	39.0
	I	5.6	45.5	4.6	7.2	37.1

## Data Availability

The data presented in this study are available on request from the corresponding author.
